# Biological functions and clinical efficacy of IL-5/IL-5Rα-targeted therapies across eosinophilia-associated diseases

**DOI:** 10.3389/fimmu.2026.1875279

**Published:** 2026-06-11

**Authors:** Johannes Lübke, Andreas Reiter, Juliana Schwaab

**Affiliations:** Department of Hematology and Oncology, University Hospital Mannheim, Heidelberg University, Mannheim, Germany

**Keywords:** benralizumab, depemokimab, eosinophilia, hypereosinophilia, interleukin-5, interleukin-5-receptor, mepolizumab, reslizumab

## Abstract

Interleukin-5 (IL-5) is a central regulator of eosinophil differentiation, maturation, survival, activation, and mobilization, and it contributes to eosinophil recruitment to inflamed tissues. These biological functions have made the IL-5/IL-5 receptor alpha (IL-5Rα) pathway a key therapeutic target in eosinophil-associated diseases. Four biologics currently target this pathway in clinical practice: mepolizumab, reslizumab, and depemokimab bind soluble IL-5, whereas benralizumab targets IL-5Rα and induces antibody-dependent cellular cytotoxicity. Clinical development has been successful in severe eosinophilic asthma (SEA), where targeting the IL-5/IL-5Rα pathway reduces exacerbation risk and can lower the need for long-term oral corticosteroid use. The therapeutic scope has since expanded to chronic rhinosinusitis with nasal polyps (CRSwNP), eosinophilic granulomatosis with polyangiitis (EGPA), and idiopathic hypereosinophilic syndrome (iHES). Mepolizumab has shown efficacy across these eosinophilia-associated diseases, reducing asthma exacerbations, nasal polyp burden, EGPA relapse activity, HES flares, and eosinophils in peripheral blood. Mepolizumab is approved by both FDA and EMA for SEA, CRSwNP, EGPA, and HES. Reslizumab improves exacerbation rates and lung function in SEA and is approved by FDA and EMA for this indication. Benralizumab produces rapid and near-complete blood and tissue eosinophil depletion, reduces exacerbation rates and oral corticosteroid use in SEA, and has demonstrated sustained remissions and corticosteroid-sparing efficacy in EGPA; it is approved by FDA and EMA for SEA and EGPA. Depemokimab extends IL-5 inhibition through a long-acting, twice-yearly dosing strategy, reduces exacerbation rates in SEA, and improves nasal polyp burden in CRSwNP; it is approved by FDA and EMA for SEA and by EMA for CRSwNP. Safety data from randomized trials, extension studies, real-world cohorts, and meta-analyses are generally reassuring, with most adverse events being mild to moderate and no consistent major safety signal. This review synthesizes current understanding of IL-5 biology, critically evaluates the clinical trial evidence for IL-5/IL-5Ra-targeted biologics across major eosinophilia-associated diseases, and highlights remaining evidence gaps and future directions.

## Introduction

Eosinophils contribute to host defense, tissue homeostasis, and immune regulation, but they can also drive inflammation, tissue injury, remodeling, and organ dysfunction. Interleukin-5 (IL-5) is a key regulator of eosinophil differentiation, maturation, survival, activation, and mobilization, and it supports eosinophil recruitment to inflamed tissues. Therefore, the IL-5/IL-5Rα pathway has become a key therapeutic target in eosinophil-associated diseases. Over the past two decades, biologics targeting this pathway have transformed the treatment of severe eosinophilic asthma (SEA) and have since been extended to chronic rhinosinusitis with nasal polyps (CRSwNP), eosinophilic granulomatosis with polyangiitis (EGPA), and idiopathic hypereosinophilic syndrome (iHES). This review summarizes key biological and clinical data on the IL-5/IL-5Rα pathway and the four agents currently used to target it in clinical practice: mepolizumab, reslizumab, benralizumab, and depemokimab.

## IL-5 biology: from cytokine structure to eosinophil lineage control

IL-5 is a homodimeric cytokine of the short-chain four-helix bundle cytokine family (1, 2). Human IL-5 is produced as a variably glycosylated, disulfide-linked homodimer of approximately 50–60 kDa, with each monomer containing 115 amino acid residues and structural features typical of the hematopoietic cytokine family (3, 4). IL-5 signals through a heterodimeric receptor composed of a ligand-specific alpha chain, IL-5Rα/CD125, and a common beta chain shared with the receptors for IL-3 and granulocyte-macrophage colony-stimulating factor (GM-CSF) (5). IL-5Rα confers ligand specificity, whereas the common beta chain mediates intracellular signal transduction (6, 7). IL-5Rα is expressed most prominently on eosinophils and eosinophil-lineage progenitors, but it is also found on basophils and mast cells (5, 8). IL-5 is more specific for eosinophils than IL-3 or GM-CSF, but the shared beta chain means that eosinophil survival and activation can also be supported through parallel cytokine pathways (9–12). This has clinical relevance because targeting the IL-5/IL-5Rα pathway may incompletely suppress tissue eosinophils in some settings, particularly where GM-CSF, IL-3, eotaxins, epithelial alarmins, or tissue survival niches remain active (13–18). Upon IL-5 binding, receptor engagement activates several intracellular signaling cascades, including JAK/STAT, Ras-MAPK, PI3K-AKT, and NF-κB-related pathways (5, 19–21). These pathways regulate eosinophil differentiation, adhesion, mediator release, and survival (10, 11, 22–24). In the bone marrow, IL-5 promotes expansion, maturation, and release of eosinophil-lineage cells (25–27). In peripheral blood, it primes eosinophils and increases responsiveness to chemotactic signals (11, 15, 28). In tissues, it prolongs survival and cooperates with chemokines such as eotaxin-1/CCL11, eotaxin-2/CCL24, and eotaxin-3/CCL26 to sustain eosinophil accumulation (13–18).

Several features make IL-5 an attractive therapeutic target. First, IL-5 acts downstream of diverse immune triggers but upstream of eosinophil expansion, activation, and tissue accumulation. Targeting this pathway can therefore modulate a shared effector mechanism across multiple eosinophilic diseases (29–31). Second, IL-5Rα is expressed predominantly on eosinophils, with more limited expression on related cell types, enabling relatively selective targeting of the eosinophil lineage, particularly with antibodies directed against IL-5Rα (5, 32). Third, eosinophils are readily quantified in peripheral blood, providing a practical biomarker for patient selection, pharmacodynamic monitoring, and response assessment (30). Fourth, clinical experience indicates that sustained eosinophil reduction or depletion is generally well tolerated, suggesting that many normal eosinophil functions can be compensated for or do not depend on high numbers of circulating eosinophils (30, 33–36). However, IL-5 is not the sole determinant of eosinophil biology. IL-5 overexpression can induce marked eosinophilia without necessarily causing organ damage, and eosinophils may persist even in IL-5-deficient settings (37–40).

## Eosinophils as effector cells, regulatory cells, and biomarkers

Eosinophils are terminally differentiated granulocytes derived from myeloid progenitors (41). Their lineage commitment is regulated by transcription factors including GATA-1, PU.1, and C/EBP family members (41, 42). Mature eosinophils contain characteristic cytoplasmic granules filled with cytotoxic proteins such as major basic protein, eosinophil cationic protein, eosinophil peroxidase, and eosinophil-derived neurotoxin (43–45). They also produce lipid mediators, reactive oxygen species, cytokines, chemokines, growth factors, and extracellular traps (46, 47). Eosinophil-derived mediators, particularly granule proteins and other pro-inflammatory products, can injure epithelium, alter airway smooth-muscle function, activate mast cells, promote procoagulant activity, and contribute to tissue remodeling and fibrosis (48–55). Yet eosinophils also have homeostatic roles. They reside in tissues under non-inflammatory conditions. Tissue-resident eosinophils may participate in immune regulation, metabolic homeostasis, and tissue repair (56–58). Although assessment of the peripheral blood eosinophil count is the most accessible biomarker for IL-5 pathway therapy, it has several limitations: It may not reflect tissue burden, fluctuate over time, or rise secondary to infections, drugs, neoplasms, adrenal insufficiency, or parasitic disease. Conversely, tissue eosinophilia can occur without marked peripheral blood eosinophilia, especially in organ-restricted eosinophilic disease (59). Therefore, additional biomarkers are being investigated to complement peripheral blood eosinophil counts. Serum IL-5 may reflect type 2 inflammation, but its clinical use is limited by low circulating concentrations, assay variability, and uncertain correlation with tissue disease activity. Eosinophil granule proteins, particularly eosinophil-derived neurotoxin (EDN), may provide a more direct measure of eosinophil activation. Serum EDN has been associated with asthma severity and asthma control, and EDN has also been evaluated as a non-invasive biomarker in eosinophilic gastrointestinal diseases, including serum and fecal EDN in pediatric eosinophilic esophagitis. However, serum IL-5, serum EDN, and fecal EDN remain investigational, and their routine use is limited by heterogeneous study designs, lack of standardized thresholds, and insufficient prospective validation against tissue eosinophilia, clinical outcomes, and response to IL-5/IL-5Rα-targeted therapy (60–66).

## Therapeutic targeting of the IL-5 pathway

Four biologics currently target the IL-5 pathway: mepolizumab, reslizumab, depemokimab, and benralizumab (**Table 1**). Mepolizumab is a subcutaneously administered, humanized IgG1κ monoclonal antibody that binds soluble IL-5. It is approved by FDA and EMA for SEA, CRSwNP, EGPA, HES, and chronic obstructive pulmonary disease (COPD). Reslizumab is an intravenously administered, humanized IgG4κ monoclonal antibody targeting soluble IL-5 which is approved by FDA and EMA for SEA. Depemokimab is an ultra-long-acting, humanized IgG1κ anti-IL-5 monoclonal antibody designed to permit extended dosing intervals; it is approved by FDA and EMA for SEA and by EMA for CRSwNP. Benralizumab is a subcutaneously administered, afucosylated, humanized IgG1κ monoclonal antibody directed against IL-5Rα. It is approved for SEA and EGPA.

**Table 1:** Approved IL-5/IL-5Rα-targeted biologics.

Biologicals (brand name)	Antibody type and mechanism of action	Administration and dosing regimen	Approval status
Mepolizumab (Nucala)	Humanized IgG1κ anti-IL-5; binds soluble IL-5 and prevents IL-5Rα engagement	SC SEA: 100 mg Q4W (adults and adolescents ≥12y) CRSwNP: 100 mg Q4W (adults) EGPA: 300 mg Q4W (adults) HES: 300 mg Q4W (adults) COPD: 100 mg Q4W (adults)	FDA/EMA: - SEA - CRSwNP - EGPA - HES - COPD
Reslizumab (Cinqair/Cinqaero)	Humanized IgG4κ anti-IL-5; binds soluble IL-5 and prevents IL-5Rα engagement	IV SEA: 3 mg/kg Q4W (adults), dose needs to be adjusted in case of significant changes in body weight	FDA/EMA: - SEA
Benralizumab (Fasenra)	Afucosylated humanized IgG1κ anti-IL-5Rα; blocks receptor signaling and induces ADCC-mediated depletion of IL-5Rα+ eosinophils/basophils.	SC SEA: 30 mg Q4W for first 3 doses, then Q8W (adults and adolescents ≥12y) EGPA: 30 mg Q4W (adults)	FDA/EMA: - SEA - EGPA
Depemokimab (Exdensur)	Long-acting humanized IgG1κ anti-IL-5 with enhanced affinity/extended half-life; binds soluble IL-5 and prevents IL-5Rα engagement.	SC SEA: 100 mg once every 6 months (twice yearly) CRSwNP: 100 mg once every 6 months (twice yearly)	FDA/EMA: - SEA - CRSwNP (approved only by EMA)

ADCC, antibody-dependent cellular cytotoxicity; COPD, chronic obstructive pulmonary disease; CRSwNP, chronic rhinosinusitis with nasal polyps; EGPA, eosinophilic granulomatosis with polyangiitis; EMA, European Medicines Agency; FDA, U.S. Food and Drug Administration; HES, hypereosinophilic syndrome; IgG, immunoglobulin G; IL-5, interleukin-5; IL-5Rα, interleukin-5 receptor alpha; IV, intravenous; Q4W, every 4 weeks; Q8W, every 8 weeks; SC, subcutaneous; SEA, severe eosinophilic asthma.

**Figure 1:**
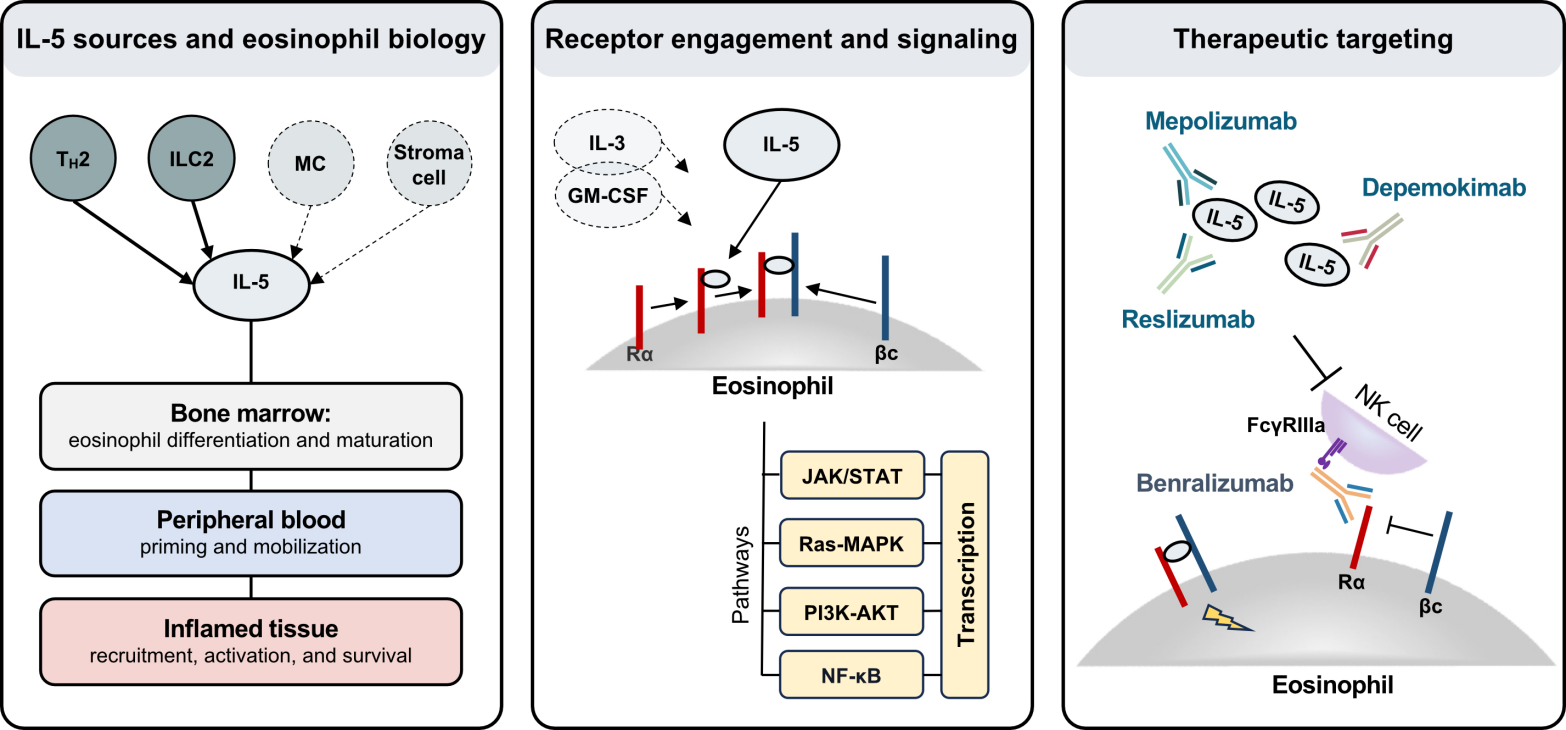
IL-5/IL-5Rα pathway and therapeutic targeting in eosinophilia-associated diseases. IL-5 is produced mainly by TH2 cells (TH2) and group 2 innate lymphoid cells (ILC2s). Under specific type 2 inflammatory conditions, additional local IL-5 production may arise from activated mast cells and stromal compartments, including airway epithelial cells and bone-marrow stromal cells. IL-5 promotes eosinophil differentiation and maturation in the bone marrow, priming and mobilization in peripheral blood, and recruitment, activation, and survival in inflamed tissues. IL-5 signals through a heterodimeric receptor composed of the ligand-specific IL-5 receptor alpha chain (IL-5Rα/CD125) and the shared common beta chain (βc/CD131). In unstimulated cells, IL-3Rα, IL-5Rα, and GM-CSFRα are present as ligand-specific monomeric receptor alpha chains. Upon ligand binding, βc is recruited to the cytokine-bound alpha-chain complex and enables downstream signal transduction. IL-5 receptor engagement activates JAK/STAT, Ras–MAPK/ERK, PI3K–AKT, and NF-κB-related signaling pathways. Mepolizumab, reslizumab, and depemokimab neutralize soluble IL-5 and prevent IL-5Rα engagement. By contrast, benralizumab binds IL-5Rα on eosinophils and induces FcγRIIIa/CD16-mediated antibody-dependent cellular cytotoxicity through natural killer cells. ILC2, group 2 innate lymphoid cell; MC, mast cell; NK cell, natural killer cell; TH2, T helper 2 cell.

Mechanistically, mepolizumab, reslizumab, and depemokimab neutralize soluble IL-5, thereby inhibiting IL-5–dependent eosinophilopoiesis, activation, and survival. By contrast, benralizumab binds IL-5Rα on eosinophils and basophils, inhibiting receptor signaling and promoting antibody-dependent cellular cytotoxicity through enhanced Fcγ receptor engagement. Accordingly, IL-5 ligand blockade is associated with reductions in circulating eosinophil counts, although tissue eosinophilia may persist. IL-5Rα targeting also leads to very reduced circulating eosinophil counts and might be associated with more pronounced reduction in tissue eosinophilia (**Figure 1**). However, direct head-to-head comparisons are lacking, and whether these pharmacodynamic differences translate into clinically meaningful differences remains uncertain.

Overall, IL-5 pathway biologics have shown a favorable safety profile. Across clinical trials and meta-analyses, no consistent major safety signal has emerged, although continued pharmacovigilance remains important as these agents are increasingly used across different eosinophil-associated diseases.

## Severe eosinophilic asthma

SEA represents a clinically important subtype of severe asthma, characterized by persistent symptoms, recurrent exacerbations, and type 2 inflammation despite high-dose inhaled corticosteroids and additional controller therapy. Although severe asthma affects only a minority of patients, it accounts for a disproportionate burden in terms of impaired quality of life, health-care use, corticosteroid exposure, and mortality (67, 68). The type 2–high inflammation is driven by cytokines such as IL-4, IL-5, IL-13, and TSLP (69) (**Table 2**).

**Table 2:** IL-5/IL-5Rα-targeting biologicals in severe eosinophilic asthma.

Biologicals (brand name)	Key studies	Key notes
Mepolizumab	DREAM	Phase 2, n=621, placebo-controlled, IV, 75/250/750 mg Q4W, lower exacerbation rate in all dosages (70)
	MENSA	Phase 3, n=576, placebo-controlled, IV (75 mg Q4W) and SC (100 mg Q4W), lower exacerbation rates in IV and SC treated patients (71)
	SIRUIUS	Phase 3, n=135, placebo-controlled, SC, 100 mg Q4W, significant corticosteroid-sparing effect (72)
	MUSCA	Phase 3b, n=551, placebo controlled, CS, 100 mg Q4W, significant improvement in quality of live (208)
	COSMEX	Phase 3b, n=652, open-label extension study, 100 mg Q4W, long-term safety and efficacy data (191)
	COLUMBA	Phase 3b, n=347, open-label extension study, 100 mg Q4W, long-term safety and efficacy data (33)
	REALTI-A	Prospective, observational cohort study, n=822, 100 mg Q4W, long-term safety and efficacy data in real-world (73, 74, 209)
*Approval status:* *− FDA: “Add-on maintenance treatment of adult and pediatric patients aged 6 years and older with severe asthma and with an eosinophilic phenotype”* *− EMA: “Add-on treatment for severe refractory eosinophilic asthma in adults, adolescents and children aged 6 years and older”*		
Reslizumab	Castro et al., 2011	Phase 2, n=106, placebo-controlled, IV, 3 mg/kg Q4W, better Asthma Control Questionnaire score (75)
	Castro et al., 2015	Two replicate phase 3 trials, n=953, placebo-controlled, IV, 3 mg/kg Q4W, lower exacerbation rates (76)
	Bjermer et al., 2016	Phase 3, n=315, placebo-controlled, IV, 0.3 or 3 mg/kg Q4W, improved FEV1, asthma control, symptoms, and quality of life (77)
	Corren et al., 2016	Phase 3, n=492, placebo-controlled, IV, 3 mg/kg Q4W, limited benefit in an unselected population, supporting eosinophil-guided use (78)
	Murphy et al., 2017	Open-label extension, n=1,051, IV, 3 mg/kg Q4W, long-term safety and efficacy data for up to 24 months (79)
	Bernstein et al., 2020	Two phase 3 trials, n=468 and n=177, placebo-controlled, SC, 110 mg Q4W, did not significantly reduce exacerbations, supports IV use (80)
*Approval status:* *− FDA: “Add-on maintenance treatment of patients with severe asthma aged 18 years and older, and with an eosinophilic phenotype”* *− EMA: “Add-on therapy in adult patients with severe eosinophilic asthma inadequately controlled despite high-dose inhaled corticosteroids plus another medicinal product for maintenance treatment”*		
Benralizumab	SIROCCO	Phase 3, n=1,205, placebo-controlled, SC, 30 mg Q4W or Q8W, reduced annual exacerbation rates and improved lung function (81)
	CALIMA	Phase 3, n=1,306, placebo-controlled, SC, 30 mg Q4W or Q8W, reduced annual exacerbation rates in severe uncontrolled asthma (82)
	ZONDA	Phase 3, n=220, placebo-controlled, SC, 30 mg Q4W or Q8W, significant oral corticosteroid-sparing effect (83)
	BORA	Phase 3 extension, n≈2,123, open-label, SC, 30 mg Q4W or Q8W, long-term safety and efficacy data (192)
	ANDHI	Phase 3b, n=656, placebo-controlled, SC, 30 mg Q8W, improved exacerbations, lung function, asthma control, symptoms, and quality of life (85)
	PONETE	Phase 3b, n≈598, open-label, SC, 30 mg Q8W, enabled elimination or substantial reduction of maintenance oral corticosteroids (86)
	MELTEMI	Long-term extension, SC, 30 mg Q8W, safety and efficacy data for up to 5 years (84)
	GREGALE	Phase 3 device study, n=116, open-label, SC, 30 mg, supported reliable pre-filled syringe administration (210)
*Approval status:* *− FDA: “add-on maintenance treatment of adult and pediatric patients aged 6 years and older with severe asthma, and with an eosinophilic phenotype”* *− EMA: “add-on maintenance treatment in adult patients with severe eosinophilic asthma inadequately controlled despite high-dose inhaled corticosteroids plus long-acting β-agonists”*		
Depemokimab	GSK3511294	Phase 1, n=61, placebo-controlled, SC, single ascending doses, extended half-life, acceptable tolerability, and sustained eosinophil reduction in asthma (87)
	SWIFT-1/-2	Phase 3, placebo-controlled, SC, 100 mg Q26W, significantly reduced annualized exacerbation rate in severe eosinophilic asthma (88)
	NIMBLE	Study in severe asthma patients previously managed with mepolizumab; evaluated switching to twice-yearly depemokimab (89)
*Approval status:* *− FDA: “Add-on maintenance treatment of severe asthma characterized by an eosinophilic phenotype in adult and pediatric patients aged 12 years and older”* *− EMA: “Add-on maintenance treatment for severe asthma with type 2 inflammation characterised by blood eosinophil count in adults and adolescents 12 years and older who are inadequately controlled despite high dose inhaled corticosteroids (ICS) plus another asthma controller”*		

This table provides a non-exhaustive overview of key studies evaluating IL-5/IL-5Rα-targeting biologicals in SEA, with emphasis on studies relevant to clinical development and regulatory approval.

ACQ, Asthma Control Questionnaire; EMA, European Medicines Agency; FDA, U.S. Food and Drug Administration; FEV1, forced expiratory volume in 1 second; ICS, inhaled corticosteroids; IV, intravenous; Q4W, every 4 weeks; Q8W, every 8 weeks; Q26W, every 26 weeks; SAE, severe asthma with eosinophilia; SC, subcutaneous.

### Mepolizumab

In DREAM (phase 2), which enrolled patients with recurrent severe exacerbations and evidence of eosinophilic inflammation, intravenous mepolizumab reduced the annual rate of clinically significant exacerbations from 2.40 per patient-year with placebo to 1.24, 1.46, and 1.15 with 75 mg, 250 mg, and 750 mg, respectively, corresponding to reductions of 48%, 39%, and 52% (70). In MENSA (phase 3), mepolizumab 100 mg subcutaneously Q4W reduced exacerbations by 53% compared with placebo, reduced exacerbations requiring emergency department visit or hospitalization by 61%, and improved forced expiratory volume in 1 second (FEV1) by approximately 98 mL versus placebo at week 32 (71). In corticosteroid-dependent SEA, SIRIUS (phase 3) demonstrated a significant oral corticosteroid-sparing effect. The median corticosteroid dose was reduced by 50% with mepolizumab versus 0% with placebo, while annualized exacerbations were reduced by 32% (72). Long-term extension studies support sustained efficacy and a favorable safety profile. In COLUMBA (phase 3b), patients were treated for up to 4.5 years, with 1201 patient-years of exposure and no new safety concerns. Exacerbation reductions were maintained over time (33). In COSMEX (phase 3b), 339 patients received mepolizumab for up to 172 weeks, adding 718 patient-years of exposure, with an on-treatment exacerbation rate of 0.93 events/year and sustained reductions in oral corticosteroid use (34). Real-world evidence further supports these findings. In REALITI-A (prospective, observational cohort study), clinically significant exacerbations decreased by 69% in an initial 1-year analysis, from 4.63 to 1.43 events/person-year, while exacerbations requiring hospitalization or emergency department visits decreased by 77% (73). In a larger REALITI-A analysis, median maintenance oral corticosteroid dose decreased by 75%, from 10.0 to 2.5 mg/day, and 43% of patients discontinued maintenance oral corticosteroid use after 1 year (74). Accordingly, mepolizumab 100 mg subcutaneously Q4W was approved by FDA and EMA.

### Reslizumab

Reslizumab, the only approved antibody administered intravenously at a dosage of 3.0 mg/kg Q4W, is an anti–IL-5 antibody with efficacy in asthma. In an early phase 2 trial of poorly controlled asthma with sputum eosinophilia, reslizumab reduced sputum eosinophils by 95% and improved FEV1 compared with placebo (75). In two replicate phase 3 studies enrolling patients with blood eosinophils ≥400/µL and prior exacerbations, reslizumab reduced clinical asthma exacerbation rate by approximately 50% and 59% versus placebo (76). Additional phase 3 data showed FEV1 improvements of up to 160 mL versus placebo over 16 weeks, with parallel improvements in asthma control and quality of life (77). Importantly, a study in patients unselected for eosinophil count failed to show significant benefit in the overall population, supporting the use of reslizumab in patients with blood eosinophils ≥400/µL (78). Long-term extension data involving 1,051 treated patients showed maintained improvements in lung function and asthma control for up to 2 years, with an acceptable safety profile (79). Fixed-dose 110 mg subcutaneous reslizumab was not effective in reducing exacerbation frequency in patients with uncontrolled asthma and increased blood eosinophils (≥300/μL), or in reducing the daily maintenance oral corticosteroid dose in patients with oral corticosteroid-dependent SEA (80). Reslizumab 3 mg/kg intravenously Q4W was approved by FDA and EMA.

### Benralizumab

In SIROCCO (phase 3), which enrolled 1,205 patients with SEA, benralizumab 30 mg subcutaneously reduced the annual asthma exacerbation rate over 48 weeks in patients with blood eosinophils ≥300/µL, with rate ratios of 0.55 for Q4W dosing and 0.49 for Q8W dosing versus placebo, corresponding to approximate reductions of 45% and 51%, respectively (81). Pre-bronchodilator FEV1 improved by 106 mL with Q4W dosing and 159 mL with Q8W dosing versus placebo. In CALIMA, a similarly designed phase 3 study enrolling 1,306 patients with SEA, benralizumab 30 mg Q4W or Q8W also significantly reduced annual exacerbation rates in patients with blood eosinophils ≥300/µL. The magnitude of exacerbation reduction was more modest than in SIROCCO (phase 3), with reported reductions of approximately 28–36% depending on dosing interval (82). In corticosteroid-dependent SEA, ZONDA established the oral corticosteroid-sparing effect of benralizumab. In this 28-week, placebo-controlled phase 3 trial of 220 patients, both benralizumab dosing regimens reduced the median maintenance oral corticosteroid dose by 75%, compared with 25% with placebo. Benralizumab also reduced overall exacerbation rates by approximately 70% and exacerbations requiring emergency department visit or hospitalization by approximately 93%, despite corticosteroid tapering (83). Longer-term studies support sustained benefit and safety. In BORA, a phase 3 extension of SIROCCO, CALIMA, and ZONDA, continued benralizumab treatment maintained reductions in exacerbations and improvements in lung function (81). In the integrated MELTEMI extension, patients previously treated in SIROCCO, CALIMA, ZONDA, and BORA were followed for up to 5 years, with long-term benralizumab reported as safe and well tolerated in severe SEA (84). ANDHI, a phase 3b trial in 656 patients, showed that benralizumab 30 mg Q8W improved exacerbation outcomes, lung function, asthma control, symptoms, and health-related quality of life in severe eosinophilic asthma (85). PONENTE, an open-label phase 3b study of 598 patients, used a personalized oral corticosteroid-reduction algorithm. The reduction phase was completed by 563 patients (94%), and approximately 63% of patient could eliminate daily oral corticosteroids, while approximately 82% eliminated oral corticosteroids or reduced to ≤5 mg/day when adrenal insufficiency limited further tapering (86). Benralizumab 30 mg subcutaneously Q4W was approved by FDA and EMA.

### Depemokimab

In the phase 1 placebo-controlled single-ascending-dose study GSK3511294, subcutaneous depemokimab showed acceptable tolerability, an extended half-life of approximately 38–53 days, and rapid, sustained reduction of eosinophils in peripheral blood. Eosinophils decreased within 24 hours after dosing, with suppression persisting for several months, supporting development of depemokimab as a twice-yearly anti–IL-5 therapy (87). The SWIFT-1 and SWIFT-2 trials established clinical efficacy in SEA. These were replicate phase 3, randomized, placebo-controlled trials in patients with SEA, prior exacerbations, and blood eosinophils ≥300/µL in the previous 12 months or ≥150/µL at screening. Patients received depemokimab 100 mg subcutaneously at weeks 0 and 26 or placebo, both added to standard care. Across both trials, 792 patients were randomized and 762 were included in the full analysis population. In SWIFT-1, the annualized exacerbation rate was 0.46 with depemokimab versus 1.11 with placebo, corresponding to a rate ratio of 0.42. In SWIFT-2, the annualized exacerbation rate was 0.56 with depemokimab versus 1.08 with placebo, corresponding to a rate ratio of 0.52. Thus, twice-yearly depemokimab reduced exacerbations by approximately 58% and 48%, respectively. Adverse-event rates were similar between depemokimab and placebo (88). NIMBLE was a randomized, double-blind, double-dummy, phase 3 non-inferiority study in patients who had documented clinical benefit from mepolizumab 100 Q4W or benralizumab 30 mg Q8W for at least 12 months. Participants were randomized to depemokimab 100 mg every 26 weeks or continued their prior biologic. Over 52 weeks, annualized clinically significant exacerbation rates were 0.57 with depemokimab and 0.49 with active comparator, with a rate ratio of 1.16; because the upper bound of the 95% confidence interval exceeded the predefined non-inferiority margin, formal non-inferiority was not met. However, exacerbation rates remained low, most participants had no clinically significant exacerbations, asthma control and lung function were stable, and adverse events were comparable between groups (89). Depemokimab 100 mg subcutaneously twice yearly was approved by FDA and EMA.

### Other treatment options

Other treatment options in severe eosinophilic/type 2 asthma include omalizumab for allergic IgE-mediated asthma, dupilumab targeting IL-4Rα and thereby IL-4/IL-13 signaling, and tezepelumab targeting thymic stromal lymphopoietin (TSLP) (90–96).

## Chronic rhinosinusitis with nasal polyps

CRSwNP is a chronic inflammatory disease of the nasal and paranasal sinus mucosa characterized by bilateral nasal polyps, nasal obstruction, rhinorrhea, loss of smell, facial pressure, sleep disturbance, and impaired quality of life (97). In Western populations, CRSwNP is frequently associated with type 2 inflammation, tissue eosinophilia, local IgE production, and increased expression of cytokines such as IL-4, IL-5, and IL-13 (98, 99). Standard treatment includes intranasal corticosteroids, short courses of systemic corticosteroids, and endoscopic sinus surgery (97). However, recurrence after surgery and repeated systemic corticosteroid exposure remain clinically important problems (100) (**Table 3**).

**Table 3:** IL-5/IL-5Rα-targeting biologicals in chronic rhinosinusitis with nasal polyps.

Biologicals (brand name)	Key studies	Key notes
Mepolizumab	Gevaert et al., 2011	Phase 2, n=30, placebo-controlled, IV, 750 mg, two doses 28 days apart; reduced nasal polyp size in a subset of patients with severe nasal polyposis (101)
	Bachert et al. ´2017	Phase 2, n=105, placebo-controlled, IV, 750 mg Q4W for 6 doses; reduced need for surgery and improved symptoms in severe recurrent nasal polyposis (102)
	SYNAPSE	Phase 3, n=407, placebo-controlled, SC, 100 mg Q4W for 52 weeks, add-on to standard of care; improved nasal polyp size and nasal obstruction in recurrent, refractory severe CRSwNP (103)
		*Post hoc* analysis of SYNAPSE, reduced nasal polyp size and nasal obstruction irrespective of comorbid asthma or AERD (104)
		*Post hoc* analysis of SYNAPSE, mepolizumab reduced the risk of further sinus surgery in patients with recurrent, refractory severe CRSwNP (105)
		Follow-up analysis after treatment discontinuation; sustained clinical benefit was reported up to 24 weeks after stopping 52 weeks of mepolizumab (106)
	MERIT	Phase 3, placebo-controlled, SC, 100 mg Q4W; evaluated mepolizumab in CRSwNP/eosinophilic chronic rhinosinusitis, supporting efficacy in eosinophilic/type 2 CRSwNP populations (107)
*Approval status:* *− FDA: “Add-on maintenance treatment of chronic rhinosinusitis with nasal polyps in adult patients aged 18 years and older with inadequate response to nasal corticosteroids.”* *− EMA: “Add-on therapy with intranasal corticosteroids for the treatment of adult patients with severe CRSwNP for whom therapy with systemic corticosteroids and/or surgery do not provide adequate disease control.”*		
Reslizumab	Gevaert et al., 2006	Phase 2, placebo-controlled, IV, single dose anti-IL-5 treatment; reduced nasal polyp size for approximately 4 weeks in about half of patients; nasal IL-5 levels predicted response (108)
Approval status: − FDA: not approved − EMA: not approved		
Benralizumab	OSTRO	Phase 3, n=413, placebo-controlled, SC, 30 mg Q4W for first 3 doses then Q8W; improved nasal polyp score and nasal blockage score, but effects on patient-reported and surgical endpoints were less consistent (109)
		Pharmacokinetic/pharmacodynamic analysis of OSTRO; demonstrated rapid and sustained depletion of eosinophils in blood and nasal polyp tissue (110)
Approval status: − FDA: not approved − EMA: not approved		
Depemokimab	ANCHOR-1/-2	Phase 3, randomized, double-blind, placebo-controlled, parallel-group trials, SC, 100 mg every 26 weeks; significantly improved co-primary endpoints of total endoscopic nasal polyp score and nasal obstruction score (111)
Approval status: − FDA: not approved − EMA: “Add-on therapy with intranasal corticosteroids for the treatment of adult patients with severe CRSwNP for whom therapy with systemic corticosteroids and/or surgery do not provide adequate disease control”		

This table provides a non-exhaustive overview of key studies evaluating IL-5/IL-5Rα-targeting biologicals in CRSwNP, with emphasis on studies relevant to clinical development and regulatory approval.

AERD, aspirin-exacerbated respiratory disease; CRSwNP, chronic rhinosinusitis with nasal polyps; EMA, European Medicines Agency; FDA, United States Food and Drug Administration; IL-5, interleukin-5; IL-5Rα, interleukin-5 receptor alpha; IV, intravenous; Q4W, every 4 weeks; Q8W, every 8 weeks; Q26W, every 26 weeks; SC, subcutaneous.

### Mepolizumab

In an early placebo-controlled study, intravenous mepolizumab 750 mg reduced nasal polyp size in a subset of patients (60%) with severe nasal polyposis (101). In the subsequent phase 2 randomized trial, 105 patients with severe recurrent nasal polyposis requiring surgery received mepolizumab 750 mg intravenously or placebo Q4W for six doses. At week 25, a significantly greater proportion of patients treated with mepolizumab no longer met criteria for surgery compared with placebo, 30% versus 10%, and mepolizumab also improved nasal polyp burden and symptoms (102). The phase 3 SYNAPSE trial established the efficacy of the currently used subcutaneous regimen in severe recurrent CRSwNP. In SYNAPSE, 407 patients were included in the intention-to-treat population and received mepolizumab 100 mg subcutaneously Q4W or placebo for 52 weeks, both added to standard of care. Mepolizumab significantly improved the total endoscopic nasal polyp score at week 52 compared with placebo, with an adjusted median difference of −0.73, and significantly improved nasal obstruction VAS score during weeks 49–52, with an adjusted median difference of −3.14 (103). Further analyses of SYNAPSE showed that mepolizumab reduced the risk of further sinus surgery in patients with recurrent, refractory severe CRSwNP and that clinical benefit could persist for up to 24 weeks after treatment discontinuation (104–106). Overall, these data support mepolizumab as an add-on treatment option in patients with severe, recurrent CRSwNP inadequately controlled by intranasal corticosteroids, systemic corticosteroids and/or surgery. The phase 3 MERIT trial extended these findings to patients with CRSwNP/eosinophilic chronic rhinosinusitis and nasal polyps from Japan, Russia, and China. In the modified intention-to-treat population, patients received mepolizumab 100 mg subcutaneously Q4W or placebo for 52 weeks, both added to standard of care. Mepolizumab significantly improved nasal obstruction VAS score from baseline to weeks 49–52 compared with placebo, with a mean treatment difference of −1.43, and showed a numerical trend toward improvement in total endoscopic nasal polyp score at week 52, with a mean treatment difference of −0.43 (107). Overall, these data support mepolizumab as an add-on treatment option in patients with severe, recurrent CRSwNP inadequately controlled by intranasal corticosteroids, systemic corticosteroids and/or surgery. Mepolizumab 100 mg subcutaneously Q4W is approved for CRSwNP by both FDA and EMA.

### Reslizumab

Reslizumab has been studied in a placebo-controlled phase 2 trial in 24 patients with bilateral nasal polyps received a single intravenous dose of reslizumab or placebo. Anti–IL-5 treatment reduced nasal polyp size for approximately 4 weeks in about half of treated patients (108). However, confirmatory data demonstrating sustained clinical efficacy in larger phase 3 trials are lacking. Accordingly, reslizumab is not approved for CRSwNP by either EMA or FDA.

### Benralizumab

The randomized, placebo-controlled phase 3 OSTRO trial enrolled 413 patients with severe CRSwNP and evaluated benralizumab 30 mg subcutaneously, given Q4W for the first three doses and Q8W thereafter. Benralizumab significantly improved the co-primary endpoints of endoscopic total nasal polyp score and patient-reported nasal blockage score at week 40 compared with placebo. The treatment difference was −0.57 for endoscopic total nasal polyp score and −0.27 for patient-reported nasal blockage score (109). Additional analyses demonstrated rapid and sustained depletion of eosinophils in blood and nasal polyp tissue, confirming pharmacodynamic activity in the upper airway compartment (110). However, treatment effects across key secondary outcomes were less consistent. Improvements in Sinonasal Outcome Test-22 (SNOT-22) score at week 40, time to first nasal polyp surgery and/or systemic corticosteroid use for nasal polyps, and time to first nasal polyp surgery did not reach statistical significance, whereas improvement in sense-of-smell score reached nominal significance (109). Consequently, despite meeting both co-primary endpoints in OSTRO, benralizumab has not obtained a CRSwNP indication from either EMA or FDA.

### Depemokimab

The phase 3 ANCHOR-1 and ANCHOR-2 trials evaluated depemokimab 100 mg subcutaneously Q26W in adults with inadequately controlled CRSwNP receiving standard of care with intranasal corticosteroids. ANCHOR-1 included 271 patients and ANCHOR-2 included 257 patients. All patients had bilateral nasal polyps with an endoscopic bilateral nasal polyp score of at least 5 and had a history of prior surgery, systemic corticosteroid use, or systemic corticosteroid intolerance. Across the pooled ANCHOR studies, depemokimab significantly improved the co-primary endpoints of total endoscopic nasal polyp score at week 52 and mean nasal obstruction score over weeks 49–52 compared with placebo. The pooled treatment difference was −0.7 for nasal polyp score and −0.24 for mean nasal obstruction score. Secondary analyses showed improvements in rhinorrhea, loss of smell, Lund-Mackay CT score, and SNOT-22, with treatment benefits observed early and maintained through week 52. Adverse-event rates were similar between depemokimab and placebo in ANCHOR-1 and ANCHOR-2, and no serious adverse events were considered treatment related (111). Depemokimab 100 mg subcutaneously twice yearly has been approved by EMA. At the time of writing, a final regulatory decision on the FDA approval of depemokimab for CRSwNP was not yet available.

### Other treatment options

Other approved biologic treatment options in severe, uncontrolled CRSwNP include dupilumab, omalizumab, and tezepelumab. These agents have demonstrated efficacy in reducing nasal polyp burden, nasal obstruction, sinonasal symptoms, and disease-related quality-of-life impairment in patients with inadequately controlled CRSwNP (112–116). Indirect comparisons suggest that dupilumab and tezepelumab may provide greater clinical benefit than IL-5/IL-5Rα–targeted biologicals for some outcomes, although these findings should be interpreted cautiously in the absence of direct head-to-head trials (117).

## Eosinophilic granulomatosis with polyangiitis

EGPA is a rare, systemic, eosinophil-rich, necrotizing granulomatous vasculitis predominantly affecting small- to medium-sized vessels and is classified among anti-neutrophil cytoplasmic antibody (ANCA)-associated vasculitis (AAV) (118–120). However, only approximately 30–47% of EGPA patients test positive for ANCA, predominantly showing anti-myeloperoxidase (MPO) ANCA positivity (121). Increasing genetic and clinical evidence indicates that ANCA-positive and ANCA-negative EGPA represent partially distinct disease subtypes, with different genetic associations and pathogenic mechanisms (122–127). ANCA-positive EGPA is more frequently associated with a vasculitic phenotype, including peripheral neuropathy, glomerulonephritis, and purpura, whereas ANCA-negative disease is more often characterized by eosinophil-driven tissue injury, particularly cardiac and pulmonary involvement. Nevertheless, ANCA status alone is not sufficient to predict individual organ involvement or to guide treatment decisions. Oral corticosteroids are the standard of care (119). In patients with severe, organ-threatening, or life-threatening disease, corticosteroid are combined with immunosuppressive agents such as cyclophosphamide, azathioprine, methotrexate or rituximab (119) (**Table 4**).

**Table 4:** IL-5/IL-5Rα-targeting biologicals in eosinophilic granulomatosis with polyangiitis.

Biologicals (brand name)	Key studies	Key notes
Mepolizumab	MIRRA	Phase 3, n=136, placebo-controlled, SC, 300 mg Q4W; significantly increased accrued weeks in remission and the proportion of patients achieving remission compared with placebo; enabled reduction in oral corticosteroid use in relapsing or refractory EGPA (128)
		*Post hoc* analyses, supported clinical benefit across baseline disease characteristics and demonstrated oral corticosteroid-sparing effects in relapsing or refractory EGPA (129)
		Open-label extension/long-term access study, SC, 300 mg Q4W; long-term mepolizumab treatment was well tolerated and associated with sustained reductions in oral corticosteroid use (130)
	Bettiol et al., 2022	Real-world observational study, n=203, SC, 300 or 100 mg Q4W; significantly reduced BVAS score, prednisone dose, and eosinophil counts from 3 months to 24 months, no significant differences between 100 and 300 mg (131)
*Approval status:* *− FDA: “*Treatment of adult patients with eosinophilic granulomatosis with polyangiitis*”* − EMA: “Add-on treatment for patients aged 6 years and older with relapsing-remitting or refractory eosinophilic granulomatosis with polyangiitis”		
Reslizumab	Manka et al., 2021	Prospective, open-label pilot study, n=10, IV, 3 mg/kg Q4W; reduced daily oral corticosteroid dose, associated with few EGPA exacerbations during treatment, favorable safety profile (132)
Approval status: − FDA: not approved − EMA: not approved		
Benralizumab	MANDARA	Phase 3, n=140, randomized, double-blind, active-controlled, non-inferiority trial, SC 30 mg Q4W; benralizumab was non-inferior to SC mepolizumab 300 mg Q4W for remission in relapsing or refractory EGPA (134)
		Benralizumab and mepolizumab were effective in reducing corticosteroids (135)
		Open-label extension/2-year analysis; patients initially treated with mepolizumab were switched to benralizumab, while those initially treated with benralizumab continued benralizumab. Longer-term follow-up showed durable remission and sustained oral corticosteroid reduction; interpretation is limited by the open-label extension design (136)
	Bettiol et al., 2023	Real-world observational study; benralizumab was associated with improvement in respiratory manifestations, reduction in disease activity, low relapse rates, and a favorable safety profile (137)
*Approval status:* *− FDA: “Treatment of adult patients with eosinophilic granulomatosis with polyangiitis”* *− EMA: “Add-on treatment for adult patients with relapsing or refractory eosinophilic granulomatosis with polyangiitis”*		
Depemokimab	Zecchin et al., 2025	Model-informed analyses support investigation of depemokimab 200 mg SC Q26W in EGPA, based on predicted sustained blood eosinophil suppression (138)
*Approval status:* *− FDA: not approved* *− EMA: not approved*		

This table provides a non-exhaustive overview of key studies evaluating IL-5/IL-5Rα-targeting biologicals in EGPA, with emphasis on studies relevant to clinical development and regulatory approval.

CI, confidence interval; EGPA, eosinophilic granulomatosis with polyangiitis; EMA, European Medicines Agency; FDA, U.S. Food and Drug Administration; IL-5, interleukin-5; IL-5Rα, interleukin-5 receptor alpha; IV, intravenous; OCS, oral corticosteroids; Q4W, every 4 weeks; Q26W, every 26 weeks; SC, subcutaneous.

### Mepolizumab

In MIRRA, a phase 3, randomized, placebo-controlled trial enrolling 136 patients with relapsing or refractory EGPA, mepolizumab 300 mg subcutaneously Q4W significantly increased remission compared with placebo. Overall, 28% of patients receiving mepolizumab achieved at least 24 weeks of accrued remission compared with 3% receiving placebo, and remission at both weeks 36 and 48 was achieved more frequently with mepolizumab than placebo. Mepolizumab also reduced relapse risk and enabled corticosteroid reduction. During weeks 48–52, 44% of mepolizumab-treated patients had an average daily oral prednisolone or prednisone dose of ≤4 mg/day compared with 7% in the placebo group (128). Subsequent *post hoc* analyses confirmed clinical benefit across baseline disease characteristics and further supported oral corticosteroid-sparing effects in relapsing or refractory EGPA (129). Long-term data from an open-label extension/long-term access study showed that continued mepolizumab 300 mg Q4W was well tolerated and associated with sustained oral corticosteroid reduction. In the long-term access cohort, 66% of patients had reduced oral corticosteroids by ≥50% at study end compared with baseline (130). Real-world evidence is consistent with these findings. In an observational study, 203 patients treated with mepolizumab 300 mg or 100 mg Q4W had significant reductions in BVAS score, prednisone dose, and eosinophil counts from 3 months through 24 months. Asthma exacerbations occurred in 82 patients overall, corresponding to 40%. By dose, exacerbations were reported in 57 of 158 patients receiving 100 mg Q4W and 17 of 33 patients receiving 300 mg Q4W, while exacerbation of the ear-nose-throat (ENT) region occurred in 31 patients, corresponding to 15%. No significant differences were observed between the 100 mg and 300 mg regimens for the reported outcomes (131). Accordingly, mepolizumab 300 mg subcutaneously Q4W was approved by FDA and EMA for EGPA.

### Reslizumab

Evidence for reslizumab in EGPA is limited. In a prospective, open-label pilot study by Manka et al., 10 patients with EGPA received reslizumab 3 mg/kg intravenously Q4W. Treatment was associated with a significant reduction in daily oral corticosteroid dose, and only 3 of 10 patients experienced an EGPA exacerbation during treatment. One patient had a severe adverse event requiring study discontinuation, but overall reslizumab was reported to have a favorable safety profile in this small cohort (132). Because of the small sample size, open-label design, and absence of a randomized comparator, these and other findings from small case series remain exploratory (133). Reslizumab is currently not approved for EGPA by either FDA or EMA.

### Benralizumab

In MANDARA, a phase 3, randomized, double-blind, active-controlled, non-inferiority trial enrolling 140 patients with relapsing or refractory EGPA, benralizumab 30 mg subcutaneously Q4W was non-inferior to mepolizumab 300 mg subcutaneously Q4W for remission. Remission at both weeks 36 and 48 was achieved in 59% of patients receiving benralizumab and 56% of patients receiving mepolizumab (134). Both treatment arms showed clinically relevant corticosteroid reduction. During weeks 48–52, complete withdrawal of oral corticosteroids was achieved in 41% of benralizumab-treated patients compared with 26% of mepolizumab-treated patients, and at least a 50% reduction in oral corticosteroid dose was reported in 86% of benralizumab-treated patients (134). In a subsequent analysis, remission off oral corticosteroids was achieved by 24% of patients receiving benralizumab compared with 11% receiving mepolizumab, corresponding to an adjusted difference of 12.5 percentage points (135). In the open-label extension and 2-year analysis, patients initially assigned to mepolizumab were switched to benralizumab, whereas those initially treated with benralizumab continued benralizumab. Longer-term follow-up showed durable remission and sustained oral corticosteroid reduction. However, interpretation is limited by the open-label extension design and the switch of the comparator arm (136). Real-world evidence further supports benralizumab activity in EGPA. In a multicentre European retrospective cohort study, 121 patients with relapsing or refractory EGPA were treated with benralizumab. Complete response, defined as BVAS 0 and prednisone dose ≤4 mg/day, increased from 12.4% at month 3 to 28.7% at month 6 and 46.4% at month 12, while partial response was observed in an additional 35.5%, 26.4%, and 18.8% of patients at the same time points (137). Accordingly, benralizumab 30 mg subcutaneously Q4W was approved by FDA and EMA for EGPA.

### Depemokimab

Depemokimab remains investigational in EGPA. Model-informed analyses by Zecchin et al. supported evaluation of depemokimab 200 mg subcutaneously Q26W in EGPA, based on predicted sustained suppression of blood eosinophils (138). The phase 3 OCEAN trial is currently evaluating the efficacy and safety of depemokimab compared with mepolizumab in adults with relapsing or refractory EGPA receiving standard-of-care therapy. Until clinical efficacy data from OCEAN become available, the role of depemokimab in EGPA remains to be defined. Depemokimab is currently not approved for EGPA by either FDA or EMA.

### Other treatment options

For life- or organ-threatening EPGA, remission induction generally consists of high-dose corticosteroids plus cyclophosphamide (119, 120). Rituximab is an option, particularly when cyclophosphamide is contraindicated, poorly tolerated, undesirable because of prior cumulative exposure or fertility concerns, or has failed (119, 120, 139–141). However, available data suggest that rituximab is more effective for vasculitis manifestations than for asthma- or ENT-dominant disease, where relapses remain frequent (119, 120, 139–141). The role of IL-5-directed therapy in remission induction is currently being investigated in a randomized, double-blind phase 3 trial comparing a mepolizumab-based regimen with conventional therapeutic strategies in patients with newly diagnosed or relapsing active EGPA (142). In non-life- or non-organ-threatening disease, methotrexate, azathioprine, or mycophenolate mofetil may be considered when IL-5/IL-5Rα-directed therapy is unavailable or as adjunctive therapy in selected patients, although evidence is limited (119, 120, 143–145). For refractory EGPA, cyclophosphamide and rituximab can be considered according to prior therapy and response (119, 120).

## Hypereosinophilia and hypereosinophilic syndromes

iHES is characterized by (i) persistent hypereosinophilia in peripheral blood (≥6 months or ≥2 weeks if end-organ damage necessitates immediate treatment), (ii) end-organ damage caused by eosinophilic infiltration and (iii) absence of a reactive, familial or neoplastic etiology, as well as exclusion of lymphocytic HES (L-HES) (146–148). L-HES is a distinct subtype, characterized by aberrant clonal T-cell populations that produce eosinophil-promoting cytokines (149). Corticosteroids remain a commonly used first-line therapy, but chronic treatment is limited by cumulative toxicity, and many patients experience recurrent flares or require additional therapy (150) (**Table 5**).

**Table 5:** IL-5/IL-5Rα-targeting biologicals in idiopathic hypereosinophilic syndrome.

Biologicals (brand name)	Key studies	Key notes
Mepolizumab	Rothenberg et al., 2008	Randomized, double-blind, placebo-controlled, n=85, IV 750 mg Q4W; reduction of prednisone to ≤10 mg/day for ≥8 consecutive weeks was achieved in 84% of patients receiving mepolizumab versus 43% receiving placebo (151)
	Roufosse et al., 2013	Open-label extension study, n=78, IV; 750 mg Q4W; supported durable disease control and acceptable long-term safety (152)
	200622	Phase 3, randomized, placebo-controlled, n=108, SC, 300 mg Q4W; reduced the proportion of patients with ≥1 HES flare or study withdrawal over 32 weeks from 56% with placebo to 28% with mepolizumab; no new safety signals were identified (153)
		*Post hoc* analysis of the phase 3 trial; flare reduction was observed irrespective of baseline HES therapy (154)
		*Post hoc* analysis of the phase 3 trial; reduced HES flares irrespective of baseline blood eosinophil count or serum IL-5 level (155)
		*Post hoc* analysis of the phase 3 trial; characterized disease flares and the clinical impact of mepolizumab on flare-related manifestations (156)
	Gleich et al., 2021	Open-label extension study, n=102, SC, 300 mg Q4W; continued control of disease flares and blood eosinophil counts, plus reductions in OCS use (157)
*Approval status:* *− FDA: “*Treatment of adult and pediatric patients aged 12 years and older with hypereosinophilic syndrome (HES) for greater than or equal to 6 months without an identifiable non-hematologic secondary cause*”* − EMA: “Add-on treatment for adult patients with inadequately controlled hypereosinophilic syndrome without an identifiable non-haematologic secondary cause”		
Reslizumab	Klion et al., 2004	Open-label pilot study, n=4, IV, reslizumab 1 mg/kg as a single dose; clinical improvement and suppression of eosinophils (158)
Approval status: − FDA: not approved − EMA: not approved		
Benralizumab	Kuang et al., 2019 Kuang et al., 2025	Phase 2, randomized, double-blind, placebo-controlled trial, n=20, SC, 30 mg Q4W for three doses followed by open-label treatment; rapid and near-complete eosinophil depletion and improved clinical outcomes (164)
		Long-term follow-up of the phase 2 trial; sustained eosinophil depletion and durable clinical benefit with acceptable safety (165)
	NATRON	Phase 3, randomized, double-blind, placebo-controlled trial, n=133, SC 30 mg Q4W for 24 weeks; reduced the risk of first HES flare by 65% versus placebo, reduced the annualized flare rate by 66%, improved fatigue, and had a safety profile comparable to placebo (166)
Approval status: − FDA: not approved − EMA: not approved		
Depemokimab	Zecchin et al., 2025	Model-informed analyses supported investigation of depemokimab 200 mg SC Q26W in iHES, based on predicted sustained blood eosinophil suppression (138)
*Approval status:* *− FDA: not approved* *− EMA: not approved*		

This table provides a non-exhaustive overview of key studies evaluating IL-5/IL-5Rα-targeting biologicals in idiopathic HES, with emphasis on studies relevant to clinical development and regulatory approval.

EMA, European Medicines Agency; FDA, U.S. Food and Drug Administration; HES, hypereosinophilic syndrome; IL-5, interleukin-5; IL-5Rα, interleukin-5 receptor alpha; IV, intravenous; Q4W, every 4 weeks; Q26W, every 26 weeks; SC, subcutaneous.

### Mepolizumab

In a randomized, double-blind, placebo-controlled trial, 85 patients with *FIP1L1::PDGFRA*-negative HES requiring corticosteroids received intravenous mepolizumab 750 mg Q4W or placebo. Reduction of prednisone to ≤10 mg/day for ≥8 consecutive weeks was achieved in 84% of patients receiving mepolizumab compared with 43% receiving placebo, demonstrating a significant corticosteroid-sparing effect (151). Long-term follow-up in an open-label extension study of 78 patients treated with intravenous mepolizumab 750 mg Q4W supported durable disease control and acceptable long-term safety (152). In a subsequent phase 3 randomized, placebo-controlled trial, 108 patients received subcutaneous mepolizumab 300 mg Q4W or placebo for 32 weeks. Mepolizumab reduced the proportion of patients with ≥1 HES flare or study withdrawal from 56% with placebo to 28% with mepolizumab, with no new safety signals (153). *Post hoc* analyses further supported the robustness of this effect, showing flare reduction irrespective of baseline HES therapy, baseline blood eosinophil count, or serum IL-5 level (154–156). In the open-label extension, 102 patients treated with subcutaneous mepolizumab 300 mg Q4W had continued control of disease flares and blood eosinophil counts, together with reductions in oral corticosteroid use (157). Accordingly, mepolizumab 300mg subcutaneously Q4W was approved by FDA and EMA.

### Reslizumab

Evidence supporting reslizumab in HES remains very limited. In an open-label pilot study, two of four patients without the *FIP1L1::PDGFRA* fusion received a single intravenous dose of reslizumab 1 mg/kg. Treatment was well tolerated. One patient achieved normalization of eosinophil counts within 48 hours, accompanied by substantial clinical improvement, with eosinophil suppression persisting for up to 12 weeks. However, eosinophilia and symptom exacerbation recurred as serum drug concentrations declined (158). Reslizumab is not approved for HES by either FDA or EMA.

In contrast, patients with myeloid/lymphoid neoplasms with *FIP1L1::PDGFRA* fusion should receive the tyrosine kinase inhibitor imatinib, which induces complete molecular remission in most patients (159–163).

### Benralizumab

In a phase 2 randomized, double-blind, placebo-controlled trial, 20 patients with symptomatic, treatment-refractory *FIP1L1::PDGFRA*-negative HES received benralizumab 30 mg subcutaneously Q4W for three doses followed by open-label treatment. Benralizumab induced rapid and near-complete eosinophil depletion and was associated with improved clinical outcomes (164). Long-term follow-up of the phase 2 cohort showed sustained eosinophil depletion and durable clinical benefit with acceptable safety (165). These findings were confirmed in NATRON, a 24-week phase 3 randomized, double-blind, placebo-controlled trial enrolling 133 patients aged ≥12 years with *FIP1L1::PDGFRA*-negative HES receiving background therapy. Benralizumab 30 mg subcutaneously Q4W reduced the risk of first HES flare by 65% compared with placebo, with flares occurring in 19.4% versus 42.4% of patients, respectively, reduced the annualized flare rate by 66% (0.41 versus 1.23 flares/year), reduced hematologic relapse by 92%, improved fatigue as assessed by PROMIS Fatigue score (mean difference –4.72), and had a safety profile comparable to placebo, with adverse events reported in 64.2% versus 66.7% of patients, respectively (166). Benralizumab is in the process of regulatory review for HES and currently not approved for HES by either FDA or EMA.

### Depemokimab

Depemokimab remains investigational in iHES. Model-informed analyses supported evaluation of depemokimab 200 mg subcutaneously Q26W in HES, based on predicted sustained suppression of blood eosinophils (138). The phase 3 DESTINY trial is evaluating depemokimab in patients with uncontrolled HES and recurrent flares. Until clinical efficacy data from DESTINY become available, the role of depemokimab in HES remains to be defined. Depemokimab is currently not approved for HES by either FDA or EMA.

### Other treatment options

In *FIP1L1::PDGFRA*-negative iHES, systemic corticosteroids are widely used for induction and flare control. In patients with corticosteroid-refractory disease or unacceptable corticosteroid toxicity, immunosuppressive and cytotoxic agents may be used, selected according to organ involvement, comorbidities, and tolerability. However, these treatments are limited by variable efficacy and cumulative toxicity.

## Pediatric evidence

Currently, pediatric evidence for IL-5/IL-5Rα-targeted biologics in SEA is most developed for mepolizumab and, more recently, benralizumab, whereas data for reslizumab and depemokimab remain substantially more limited. Mepolizumab is approved by both the FDA and EMA as add-on maintenance therapy for SEA in children aged ≥6 years, with a recommended dose of 40 mg every 4 weeks in children aged 6–11 years and 100 mg every 4 weeks in patients aged ≥12 years. This pediatric indication was initially supported by extrapolation from adult and adolescent phase II/III trials, in which adolescents represented only a small proportion of the overall population, and by an open-label pediatric study in 36 children aged 6–11 years showing pharmacokinetic/pharmacodynamic exposure in younger patients, reduction of peripheral blood eosinophils, and safety findings broadly consistent with adult experience (167–169). In a randomized placebo-controlled trial specifically enrolling children and adolescents with exacerbation-prone eosinophilic asthma, mepolizumab reduced the rate of severe exacerbations over 52 weeks, although improvements in asthma control, lung function, and fractional exhaled nitric oxide were not significant (170). Mepolizumab is also approved by the FDA for the treatment of pediatric patients aged ≥12 years with HES, and by the EMA for patients aged ≥6 years with relapsing-remitting or refractory EGPA. Benralizumab has also moved into the pediatric setting. Earlier phase III asthma trials included only small adolescent subgroups, limiting firm efficacy conclusions in patients aged 12–17 years (171). However, an open-label study in children aged 6–11 years demonstrated pharmacokinetic, pharmacodynamic, and safety findings consistent with previous adolescent and adult data, including significant blood eosinophil depletion (172). On this basis, benralizumab was approved by the FDA for severe asthma with an eosinophilic phenotype in patients aged ≥6 years, with a dose of 10 mg for children aged 6–11 years weighing <35 kg and 30 mg for those weighing ≥35 kg, whereas the EMA indication remains restricted to adults. Reslizumab is not approved by either the FDA or EMA for use in children or adolescents. Although early clinical studies included small numbers of adolescents with severe eosinophilic asthma, the available data were insufficient to establish pediatric efficacy or to support regulatory approval in this population (76). In the phase III SWIFT-1 and SWIFT-2 trials of depemokimab in patients aged ≥12 years with severe asthma and an eosinophilic phenotype, adolescents were also underrepresented, accounting for only 30 of 792 enrolled patients aged 12–17 years (88). Moreover, no clinical experience is currently available in children younger than 12 years.

## Other eosinophilic and allergic diseases

IL-5/IL-5Rα-targeting antibodies have also been evaluated in eosinophilic and allergic diseases beyond SEA, CRSwNP, EGPA, and HES, but efficacy has been more heterogeneous. In eosinophilic esophagitis, mepolizumab, reslizumab, and benralizumab reduced eosinophils in peripheral blood and/or esophageal eosinophilia in several trials, but clinical benefit was inconsistent, and reductions in peak esophageal eosinophil counts did not reliably translate into meaningful improvement in dysphagia, endoscopic disease activity, or patient-reported outcomes (173–178). Similarly, in eosinophilic gastritis and other eosinophilic gastrointestinal diseases, benralizumab induced near-complete depletion of eosinophils in peripheral blood or gastrointestinal tissue biopsies, but symptom improvement was variable (179, 180). In atopic dermatitis, mepolizumab reduced eosinophils in peripheral blood and, in allergen-challenged skin, decreased eosinophils in skin, but this did not translate into consistent improvement in eczema severity, atopy patch test responses, or clinical disease activity (181–184). In chronic spontaneous urticaria, benralizumab showed early signals in a small study, whereas a larger phase IIb trial did not demonstrate a meaningful effect. Isolated reports have also described worsening of urticaria during benralizumab treatment (185–188). For other eosinophilic disorders, including allergic bronchopulmonary aspergillosis, eosinophilic pneumonia, drug-induced eosinophilic disease, and selected dermatologic eosinophilic syndromes, evidence is largely limited to case reports, small cohorts, or observational studies (189, 190).

## Safety

Across indications, IL-5/IL-5Rα-targeting antibodies have shown a generally favorable safety profile, with most adverse events being mild to moderate and broadly similar to placebo or active-comparator groups. For mepolizumab, the most frequently reported adverse events include headache, nasopharyngitis, upper respiratory tract infection, bronchitis, injection-site reactions, back pain, and asthma-related events. Long-term studies in SEA did not identify new safety signals over several years of exposure, and long-term studies in EGPA and HES also supported sustained tolerability despite prolonged eosinophil suppression (34, 70, 73, 103, 128, 130, 131, 136, 152, 153, 157, 191). For reslizumab, adverse events in asthma trials were generally comparable with placebo, with asthma worsening, nasopharyngitis, upper respiratory tract infection, headache, and sinusitis among commonly reported events. Because reslizumab is administered intravenously, infusion-related reactions and hypersensitivity are clinically relevant. Anaphylaxis has been reported. Experience with reslizumab outside SEA remains limited (76–80). In HES, reslizumab was well tolerated in four patients, but eosinophilia and symptom exacerbation occurred as drug levels waned (158). For benralizumab, common adverse events include headache, pharyngitis, nasopharyngitis, upper respiratory tract infection, injection-site reactions, and asthma-related events. Long-term SEA extension studies reported sustained tolerability for up to 5 years without a consistent signal for increased serious infection, malignancy, or other major complications despite near-complete eosinophil depletion (35, 81, 109, 192). In EGPA and HES, long-term studies supported acceptable safety (134, 137, 166). For depemokimab, pooled analyses supported twice-yearly depemokimab as generally well tolerated over 52 weeks (88, 111, 193). Switching from mepolizumab or benralizumab to twice-yearly depemokimab was associated with comparable adverse-event rates (89). A meta-analysis by Li et al. included 24 studies of mepolizumab, reslizumab, and benralizumab, and found that the safety profiles of individual anti-IL-5/IL-5Rα agents were broadly consistent with their respective prescribing information. Commonly reported adverse events included headache, nasopharyngitis, upper respiratory tract infection, bronchitis, and worsening of asthma. No major safety signal emerged from randomized trial data (194). Gleich et al. summarized evidence from patients with spontaneous or acquired eosinophil deficiency, eosinophil-deficient mouse models, and anti-IL-5-treated patients, concluding that absence or marked reduction of eosinophils has not been associated with a consistent pattern of impaired health under usual conditions (36).

## Evidence gaps

Special populations remain underrepresented in IL-5/IL-5Rα trials. Pediatric data are strongest for severe eosinophilic asthma, particularly for mepolizumab, where pharmacokinetic, pharmacodynamic, and safety studies in children aged 6–11 years showed substantial reduction in eosinophils and a safety profile broadly consistent with that observed in older patients (168, 169). Pediatric evidence in CRSwNP, EGPA, HES and other eosinophilic diseases remains more limited and often extrapolated from adult studies. Older adults have also been underrepresented in randomized trials, although real-world asthma cohorts suggest that anti-IL-5/IL-5Rα treatment can be effective and generally well tolerated in elderly patients. The interpretation is however limited by comorbidity, polypharmacy, infection risk, and selection bias (195, 196).

Pregnancy data remain sparse and consist mainly of case reports or case series. Successful pregnancies have been reported during exposure with mepolizumab or benralizumab, but available data are insufficient to define fetal, neonatal, or maternal risk (197–199). Patients with active malignancy, immunodeficiency, chronic infection, helminth exposure, or complex hematologic disease were commonly excluded from trials and require individualized risk assessment.

An additional evidence gap concerns the relationship between blood eosinophil depletion, tissue eosinophilia, and organ-specific clinical response. Clinical responses may be heterogeneous across organ systems within the same patient, suggesting that suppression of peripheral eosinophilia does not necessarily translate into uniform control of all organ manifestations. This is particularly relevant because eosinophil-associated diseases often combine eosinophil-driven pathology with variable degrees of broader type 2 or non–type 2 inflammation. Consequently, some patients require persistent background therapy despite biologic treatment, including systemic corticosteroids, topical or inhaled corticosteroids, immunosuppressive agents such as methotrexate, azathioprine or cyclophosphamide, or other organ-directed therapies. The extent and durability of oral corticosteroid reduction are therefore heterogeneous, and steroid-sparing effects observed at trial population level may not reliably predict successful tapering in individual patients or across different eosinophilia-associated entities.

Treatment discontinuation is another unresolved area. In the randomized COMET study, patients with severe eosinophilic asthma who stopped long-term mepolizumab had more exacerbations and worse asthma control than those who continued therapy, and related analyses showed shorter time to first asthma worsening after withdrawal (200, 201). Studies on discontinuation of biologicals in severe asthma suggest that stopping biologics is feasible only in a subset of patients with sustained, well-controlled disease. Proposed criteria include an absence of asthma symptoms (asthma control questionnaire score <1.5 or asthma control test score >19), no asthma exacerbations, no use of oral corticosteroids, normalized spirometry (FEV1 ≥80%), suppressed T2 inflammation (blood eosinophils in peripheral blood < 0.3 x10^9^/L and fractional exhaled nitric oxide <50 ppb), and control of asthma comorbidities. However, current evidence is dominated by data on omalizumab and mepolizumab, whereas data for benralizumab and reslizumab remain limited to observational analyses or individual cases (202). Furthermore, their applicability to other entities remains undefined.

IL-5/IL-5Rα-targeted biologics are expensive, and their cost-effectiveness depends on multiple factors, including drug price, baseline exacerbation risk, oral corticosteroid exposure, health-care utilization, quality-of-life gains, and local willingness-to-pay thresholds. Economic analyses in SEA have therefore yielded variable results. Some models suggest limited cost-effectiveness at current prices, whereas others support their use in carefully selected high-risk populations, particularly when reductions in exacerbations, hospitalizations, and corticosteroid-related morbidity are considered (203–207). Health-economic data are even more limited for non-asthma indications such as CRSwNP, EGPA, and HES, where patient numbers are smaller, outcomes differ, and comparisons between mepolizumab, benralizumab, reslizumab, and depemokimab remain indirect. Further studies are therefore needed to better define the value of IL-5/IL-5Rα-targeted therapy across eosinophilia-associated diseases.

## Conclusion

The central role of IL-5 in eosinophil biology explains the clinical efficacy of IL-5/IL-5Rα-targeted therapies. The strongest clinical evidence is in SEA. However, data in EGPA and HES show that blocking the IL-5/IL-5Rα pathway can also reduce systemic disease activity and corticosteroid exposure. Clinical experience has also defined the limits of this pathway. Blood or tissue eosinophilia does not, by itself, establish IL-5 dependence. Likewise, eosinophil reduction does not always translate into symptom control. This is evident in eosinophilic esophagitis, atopic dermatitis, and chronic spontaneous urticaria. In these diseases, IL-5/IL-5Rα-targeted therapy can reduce eosinophils, but clinical benefit is inconsistent. The key therapeutic question is therefore not only whether eosinophils are present. It is whether eosinophils are dominant effector cells that drive the disease. Future progress will require more precise patient selection, better biomarkers, and prospective data on the comparison of different biologicals and how to sequence them. Tissue-based profiling, single-cell sequencing, spatial transcriptomics, proteomics, and other high-resolution approaches may help define IL-5-dependent entities and identify patients most likely to benefit from blocking the IL-5/IL-5Rα pathway. Such a precision medicine approach will be essential to maximize the clinical benefit of these powerful but expensive therapies.
